# Comparative analysis of the gut microbiota composition and diversity in *Erinaceus amurensis* from the Wandashan Mountain range area based on metagenomics

**DOI:** 10.3389/fmicb.2024.1479352

**Published:** 2024-12-05

**Authors:** Xiuqi Jia, Qiang Li, Xinyu Yang, Dianwei Li, Zhimin Jin

**Affiliations:** College of Life Science and Technology, Mudanjiang Normal University, Mudanjiang, China

**Keywords:** *Erinaceus amurensis*, metagenomics, gut microbiota, diversity, comparative analysis

## Abstract

This study aimed to preliminarily explore the composition and diversity of intestinal bacteria in *Erinaceus amurensis* during breeding period, aiding in the field rescue and population conservation efforts of *Erinaceus amurensis*. This also provides foundational data for further research on the prevention and screening of Emerging Zoonotic Infectious Diseases and the experimental animalization of wild *Erinaceus amurensis*. Between April and July 2023, we collected 13 fresh fecal samples from *Erinaceus amurensis* at the Sishan Forest Farm in Jidong County, Heilongjiang Province, situated within the Wandashan Mountain range. Utilizing metagenomic sequencing technology, we conducted a comparative analysis of the gut microbiota composition and diversity in wild *Erinaceus amurensis* across different genders and between adult and fetal individuals within the same habitat. Our results revealed significant differences (*P* < 0.01) in the classification and diversity of gut microbiota between genders and between adult and fetal *Erinaceus amurensis*. Specifically, the dominant bacterial groups in the gut of *Erinaceus amurensis* were *Pseudomonas, Proteobacteria*, and *Enterobacteriaceae*. In male and female *Erinaceus amurensis*, the dominant bacterial groups were *Pseudomonas, Bacteroides*, and *Firmicutes*, with variations in bacterial abundance and diversity. While male and female *Erinaceus amurensis* exhibited similar microbial compositions, they displayed significant differences in specific bacterial classifications. The dominant bacterial group in fetal *Erinaceus amurensis* was *Proteobacteria*, which demonstrated lower diversity and abundance compared to the adult group. Furthermore, the types and abundance of pathogenic or opportunistic pathogens in the gut of fetal *Erinaceus amurensis* and male *Erinaceus amurensis* were higher than those in female *Erinaceus amurensis*. The analysis of experimental results indicates that *Erinaceus amurensis* in this region either have or are at risk of developing inflammation related to the intestinal and urinary tracts, as well as skin-related issues. Consequently, it is advised that forestry and wildlife conservation personnel in this area prioritize treatment against these specific pathogens when conducting rescue operations for *Erinaceus amurensis* in the wild.

## 1 Introduction

The gut microbiota, a vast community of microbes within the animal body, plays an essential role in various aspects of the host's physiological activities and behaviors (Schretter, 2020; Greenhill, 2017). Microbial community aids the host animal in adapting to climate and environmental changes, as well as pathogens in nature, by influencing functions such as digestion, absorption, and excretion (Yeoman et al., 2011; Zhu et al., 2011), metabolism (Eckel, 2021), immunity (Yoo et al., 2020), nutrition, and development (Fu et al., 2024). Concurrently, the health status of the organism and various environmental factors can significantly influence the gut microbiota of the host animal. This, in turn, can have a profound impact on the related behaviors exhibited by the animal (Sepulveda and Moeller, 2020). Over the course of biological evolution, the gut microbiota has gradually formed an integrated whole through mutual symbiosis and interaction.

*Erinaceus amurensis*, an Insectivora of the *Erinaceidae* family and *Erinaceus* genus (Bai et al., 2022), is extensively distributed across various provinces and regions in China. Its habitat primarily encompasses forests, shrublands, grasslands, farmlands, and orchards. The nocturnal species burrows in shrubs, tree roots, and crevices. Its diet comprises insects, rodents, birds, small animals, as well as fruits and legumes. It hibernates and is one of the three protected species in China (Cassola, 2016; Şeker, 2024). The *Erinaceus amurensis* possesses several advantages including its wide distribution in the wild, moderate size, ease of capture, high offspring rate, docile temperament, and low aggressiveness, making it a potential experimental animal. However, its valuable fur for traditional Chinese medicine research has led to extensive hunting in the wild. Coupled with its limited ability to defend against natural enemies, its population in the wild is declining annually.

As sequencing technology continues to evolve, numerous studies have been conducted on the gut microbial communities of humans, economic animals, and wild animals (Belda et al., 2022; Levin et al., 2021; Liu et al., 2024; Li et al., 2024). Nevertheless, research on *Erinaceus amurensis* has been primarily limited to basic physiology, biochemistry, plumpness, hypoxia tolerance, karyotype analysis, and molecular epidemiological surveys (Lanave et al., 2023; Zhang et al., 2021; Yang et al., 1991). To date, there have been no reports on the gut microbiota of *Erinaceus amurensis* in their natural habitat. In this study, we employed metagenomic sequencing technology to compare and analyze the composition and diversity of gut microbiota between different genders and between fetuses and adults in the Wandashan Mountain range. Our aim is to provide a theoretical foundation for the protection of wild *Erinaceus amurensis* populations and related experimental animal research. Additionally, our findings may aid in the early detection of new zoonotic infectious diseases. This research will contribute fundamental scientific data to the fields of microbiology, ecology, and veterinary medicine, thereby advancing public health initiatives. Humans have a long-standing history of utilizing wildlife, which has significantly evolved over thousands of years. A substantial portion of this utilization involves the application of “animal medicine.” The skin of the spiny *Erinaceus amurensis*, a valuable traditional Chinese medicinal material, holds significant research potential in the field of traditional Chinese medicine. Studying the distribution and diversity of gut microbiota in *Erinaceus amurensis* can provide fundamental scientific data for related research on their domestication. For example, the types and distribution of gut microbiota can inform the development of specific feed for *Erinaceus amurensis* and facilitate research into targeted vaccines related to the health relationship between gut microbes and their hosts.

## 2 Materials and methods

### 2.1 Study area and sampling

Based on the nocturnal activity patterns of the *Erinaceus amurensis*, a cage trapping method was designed and used from April to July 2023 in Sishan Forest Farm (131°54′47″E−131°8′18″, 44°58′53″−44°51′12″N) in Jidong County, Heilongjiang Province. Total of 13 *Erinaceus amurensis* were captured, including five females, five males, and three fetuses. The samples were divided into female (FM), male (M), and fetus (FE) groups. Fresh and complete mid-section fecal samples were collected using noninvasive sampling methods (Fasoli et al., 2021), placed in sterile plastic bags for classification and labeling, and transported to the laboratory on dry ice. After labeling, they were stored in an ultra-low temperature freezer at −80°C temporarily.

### 2.2 DNA extraction, library construction, and metagenomic sequencing

Total genomic DNA was extracted from fecal samplessamples using the Mag-Bind^®^ Soil DNA Kit (Omega Bio-tek, Norcross, GA, U.S.) according to manufacturer's instructions. Concentration and purity of extracted DNA was determined with TBS-380 and NanoDrop2000, respectively. DNA extract quality was checked on 1% agarose gel.

DNA extract was fragmented to an average size of about 400 bp using Covaris M220 (Gene Company Limited, China) for paired-end library construction. Paired-end library was constructed using NEXTFLEX Rapid DNA-Seq (Bioo Scientific, Austin, TX, USA). Adapters containing the full complement of sequencing primer hybridization sites were ligated to the blunt-end of fragments. Paired-end sequencing was performed on Illumina NovaSeq (Illumina Inc., San Diego, CA, USA) at Majorbio Bio-Pharm Technology Co., Ltd. (Shanghai, China) using NovaSeq 6000 S4 Reagent Kit v1.5 (300 cycles) according to the manufacturer's instructions (www.illumina.com).

### 2.3 Sequencing data

Raw data have been submitted to NCBI (accession number: SUB14652556).

### 2.4 Sequence quality control and genome assembly

The data were analyzed on the free online platform of Majorbio Cloud Platform (www.majorbio.com). Briefly, the paired-end Illumina reads were trimmed of adaptors, and low-quality reads (length < 50 bp or with a quality value < 20 or having N bases) were removed by fastp (Chen et al., 2018) (https://github.com/OpenGene/fastp, version 0.20.0).

Reads were aligned to the *Erinaceus amurensis* Mitochondrial genome by BWA (Li and Durbin, 2009) (http://bio-bwa.sourceforge.net, version 0.7.9a) and any hit associated with the reads and their mated reads were removed.

Metagenomics data were assembled using MEGAHIT (Li et al., 2015) (https://github.com/voutcn/megahit, version 1.1.2), which makes use of succinct de Bruijn graphs. Contigs with a length ≥300 bp were selected as the final assembling result, and then the contigs were used for further gene prediction and annotation.

### 2.5 Constructing non-redundant gene set

A non-redundant gene catalog was constructed using CD-HIT (Fu et al., 2012) (http://www.bioinformatics.org/cd-hit/, version 4.6.1) with 90% sequence identity and 90% coverage. High-quality reads were aligned to the non-redundant gene catalogs to calculate gene abundance with 95% identity using SOAPaligner (Li et al., 2008) (http://soap.genomics.org.cn/, version 2.21).

### 2.6 Species and functional annotation

The amino acid sequences from the non-redundant gene set were aligned with the NR database utilizing Diamond (Buchfink et al., 2015) (http://diamondsearch.org/forums/index.php, version 0.8.35) (with BLASTP alignment parameter settings and an expected value e-value of 1e-5). Subsequently, species annotations were procured from the corresponding taxonomy information database within the NR library. The abundance of each species was then determined by summing the gene abundances associated with that particular species.

### 2.7 Complexity analysis

An analysis of Alpha Diversity was performed on three distinct sample sets to compare the diversity differences in the gut microbiota of male and female *Erinaceus amurensis*, as well as fetuses *Erinaceus amurensis*. An analysis of Alpha Diversity was performed on three distinct sample sets to compare the diversity differences in the gut microbiota of male and female *Erinaceus amurensis*, as well as fetuses *Erinaceus amurensis*. The ACE and Chaol indices serve as indicators of microbial community richness, while the Simpson and Shannon indices reflect community diversity. A larger Chao1 index and ACE index suggest a greater total number of species, with higher community diversity indicated by a larger Shannon index and a smaller Simpson index (Eckburg et al., 2005). A one-way analysis of variance (ANOVA) and Tukey's test were conducted on the Alpha diversity indices of the three sample sets to compare intergroup correlations and differences, with *P* < 0.05 considered statistically significant and *P* < 0.01 extremely significant. Non-metric multidimensional scaling (NMDS) and analysis of similarities (ANOSIM) were employed to analyze the composition of the microbial communities. NMDS can illustrate the degree of difference between different samples through the distance between points, with closer distances indicating more similar species compositions. A stress value < 0.2 indicates that there are differences between samples (Kilroy et al., 2020). ANOSIM can test for both intergroup and intragroup differences, with *R* values ranging from −1 to 1. An *R* value >0 indicates that intergroup differences are greater than intragroup differences; otherwise, intergroup differences are less than intragroup differences. A *P*-value < 0.05 indicates statistical significance (Clarke et al., 2016).

### 2.8 Bacterial community typing analysis

The R language's ade4 package, cluster package, and clustersim package are utilized to compute the Jensen-Shannon Distance and PAM (Partitioning Around Medoids) for clustering based on the relative abundance of microbial communities at the selected taxonomic level. The optimal clustering *K* value is ascertained by the Calinski-Harabasz (CH) index, which is subsequently visualized using Between-class analysis (BCA, *K* ≥ 3) or principal coordinates analysis (PCoA, *K* ≥ 2) (Noval Rivas et al., 2013). This methodology is employed to statistically analyze the typing of dominant microbial community structures in various samples.

## 3 Results and analysis

### 3.1 Comparative analysis of gut microbiome composition and species abundance in *Erinaceus amurensis* from Wandashan Mountain range area

**Figure 1:**
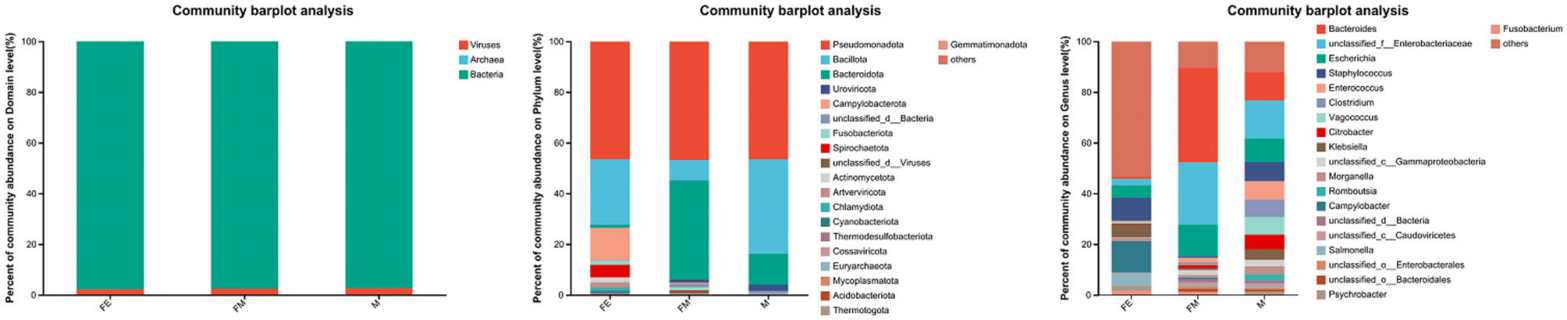
Gut microbial composition of the *Erinaceus amurensis* in Wandashan mountains. The left side is the Domain level, the middle is the Phylum level, the right side is the genus level. FM, female; M, male; FE, fetus.

The 13 fecal samples collected from the *Erinaceus amurensis* were categorized at the domain level. Of these, bacteria constituted the most significant proportion, with a relative kurtosis of 97.80%. This was followed by viruses, which accounted for a mere 2.19% of the total. The least abundant group was archaea, with a relative abundance of just 0.01% (Figure 1).

At the phylum classification level, a total of 79 gut microbiota were identified in the *Erinaceus amurensis* from Wandashan Mountain range area. These included five groups with an abundance exceeding 1%, namely *Pseudomonadota* (46.43%), *Bacillota* (37.25%), *Bacteroidota* (12.2%), *Uroviricota* (2.49%), and Unclassified-Bacteria (1.12%). In the FM group, seven groups had an abundance exceeding 1%, including *Pseudomonadota* (46.73%), *Bacteroidota* (39.1%), *Bacillota* (8.03%), Unclassified-Bacteria (1.26%), *Fusobacteriota* (1.25%), *Uroviricota* (1.23%), and Unclassified Viruses (1.04%). The FE group contained nine groups with an abundance exceeding 1%, specifically *Pseudomonadota* (46.39%), *Bacillota* (25.84%), *Campylobacterota* (12.58%), *Spirochaetota* (4.95%), *Artverviricota* (2.07%), *Actinomycetota* (1.98%), *Fusobacteriota* (1.72%), *Chlamydiota* (1.23%), and *Bacteroidota* (1.22%).

**Figure 2:**
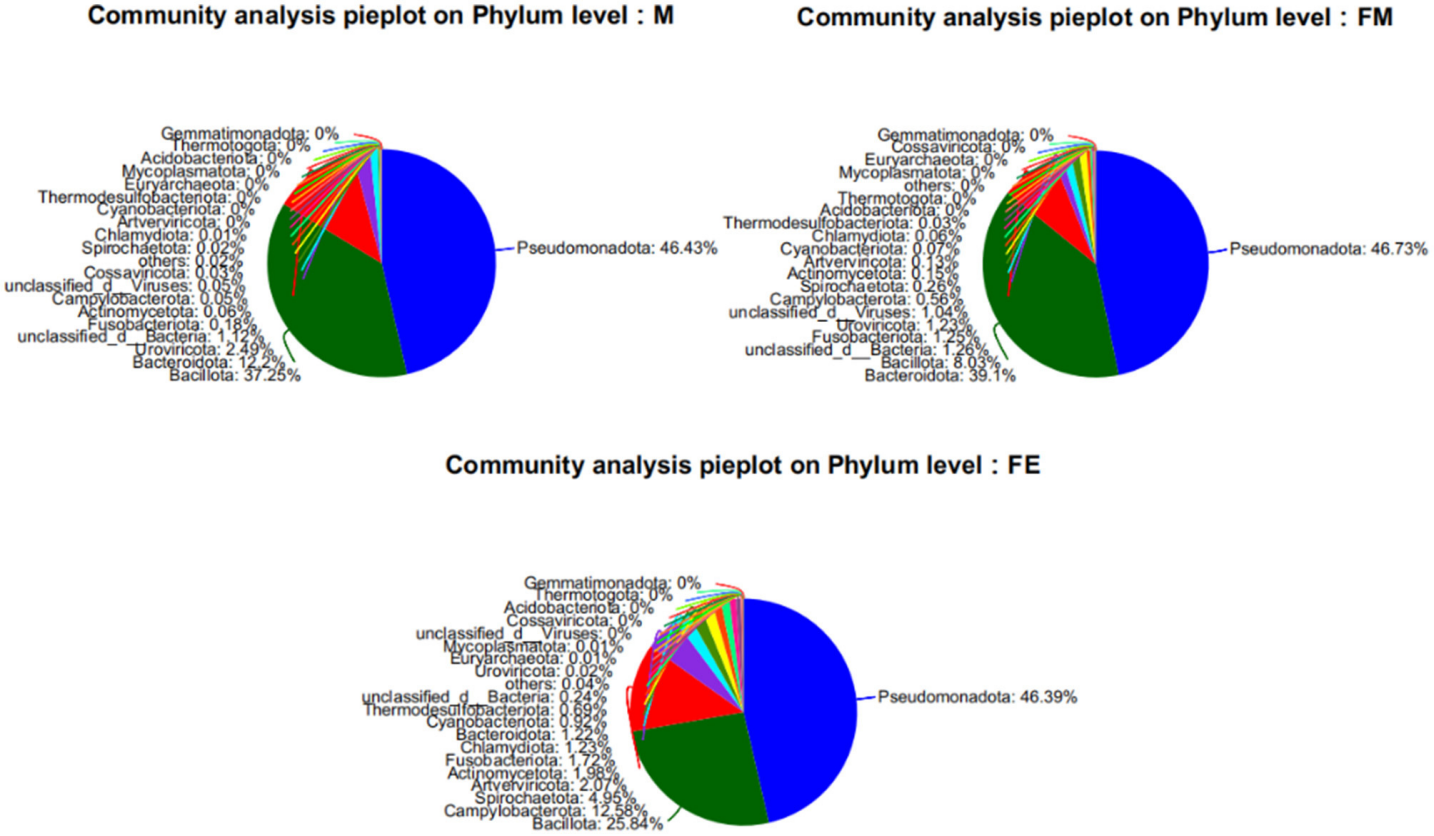
Relative abundance of samples at the phylum level of gut microbes in each group. FM, female; M, male; FE, fetus.

The *Pseudomonadota* phylum was the most dominant in three distinct groups. The abundance of *Bacillota* was significantly higher in both the M and FE groups compared to the FM group. Among the three groups, the FE group exhibited the lowest abundance of *Bacteroidota*, constituting only 1.22% (Figure 2).

**Figure 3:**
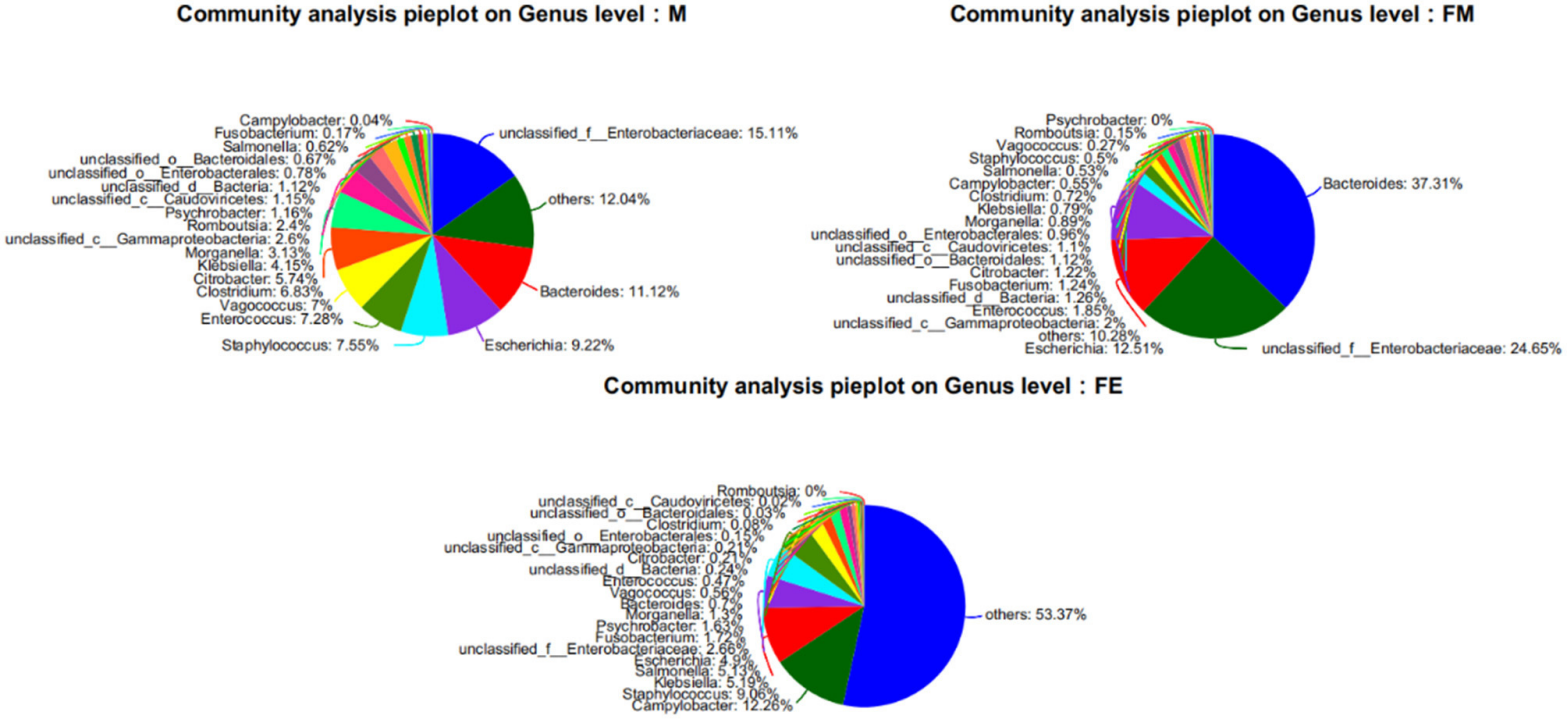
Relative abundance of samples at the genus level of gut microbes in each group. FM, female; M, male; FE, fetus.

At the genus level, a total of 1,416 microorganisms were identified across three groups. In group M, 15 genera had an abundance exceeding 1%, including Unclassified-f-*Enterobacteriaceae* (15.11%), Others (12.04%), *Bacteroides* (11.12%), *Escherichia* (9.22%), *Staphylococcus* (7.55%), *Enterococcus* (7.28%), *Vagococcus* (7%), *Clostridium* (6.83%), *Citrobacter* (5.74%), *Klebsiella* (4.15%), *Morganella* (3.13%), Unclassified-c-*GammaProteobacteria* (2.6%), *Romboutsia* (2.4%), *Psychrobacter* (1.16%), Unclassified-c-*Caudoviricetes* (1.15%), and Unclassified-c-Bacteria (1.12%). In group FM, 11 genera had an abundance greater than 1%, namely *Bacteroides* (37.31%), Unclassified-f-*Enterobacteriaceae* (24.65%), *Escherichia* (12.51%), Others (10.28%), Unclassified-c-*GammaProteobacteria* (2%), *Enterococcus* (1.85%), Unclassified-c-*Bacteria* (1.26%), *Fusobacterium* (1.24%), *Citrobacter* (1.22%), Unclassified-o-*GammaProteobacteria* (1.12%), and Unclassified-c-*Caudoviricetes* (1.1%). In group FE, 10 genera had an abundance exceeding 1%, specifically Others (53.37%), *Campylobacter* (12.26%), *Staphylococcus* (9.06%), *Klebsiella* (5.19%), *Salmonella* (5.13%), *Escherichia* (4.9%), Unclassified-f-*Enterobacteriaceae* (2.66%), *Fusobacterium* (1.72%), *Psychrobacter* (1.63%), and *Morganella* (1.3%). The genus Bacteroides was the most abundant in both group M and FM, but its relative abundance in group FE was only 0.7% (Figure 3).

**Figure 4:**
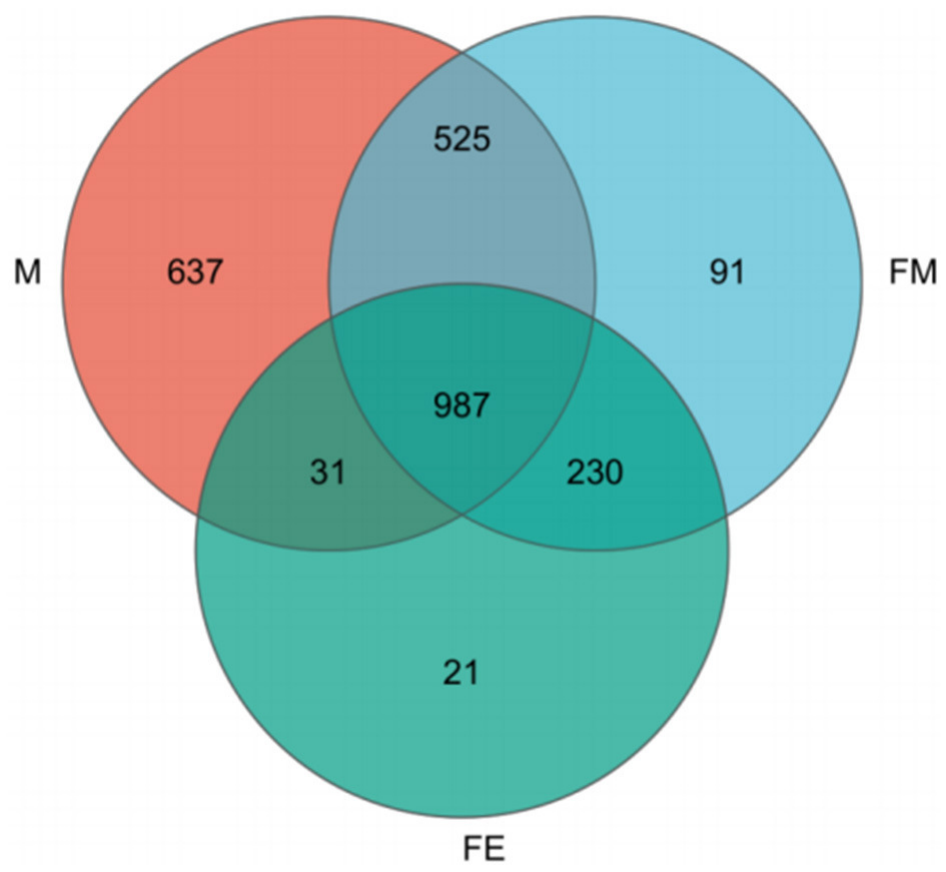
Annotation Map of NR species across three taxonomic levels. FM, female; M, male; FE, fetus.

The NR species annotation map reveals that Group M encompasses 637 unique bacterial communities, while Groups FM and FE contain 91 and 21 unique bacterial communities respectively. Furthermore, there are 525 shared bacterial communities between Groups FM and M, 31 between Groups M and FE, and a substantial 230 between Groups FM and FE. In total, the three groups share 987 common bacterial communities (Figure 4).

### 3.2 Comparative analysis of intestinal microbiota diversity in *Erinaceus amurensis* from Wandashan Mountain range area

**Figure 5:**
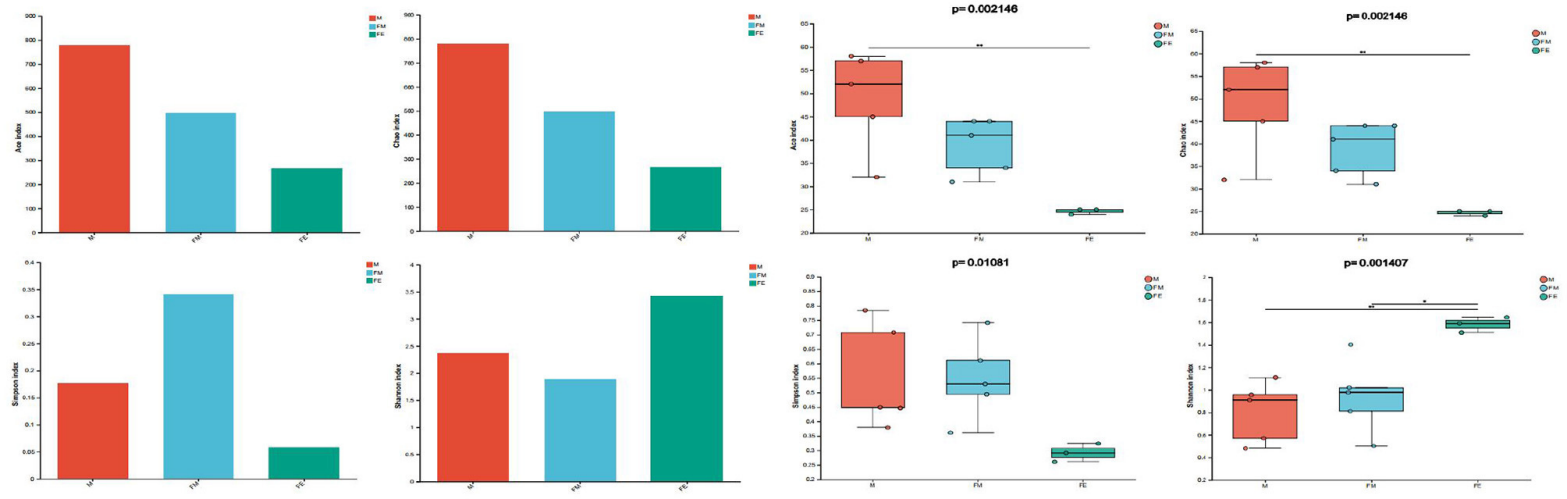
Diversity and differences of gut microbiota in three groups. FM, female; M, male; FE, fetus.

The complexity analysis, specifically Alpha Diversity, revealed significant differences in the abundance (Chao1 and Ace) and evenness (Simpson and Shannon) of gut microbiota at the genus level among three groups (*P* ≤ 0.01). The Chao1 and Ace values were higher in males (M group) compared to females (FM group) and fetuses (FE group), with the lowest values observed in the fetal (FE) group. This suggests that the gut microbiota abundance was least in the fetal (FE) group. The Simpson index and Shannon index analyses yielded consistent results: the evenness of gut microbial communities was higher in fetuses (FE group) than in males (M group), while it was lowest in females (FM group; Figure 5).

**Figure 6:**
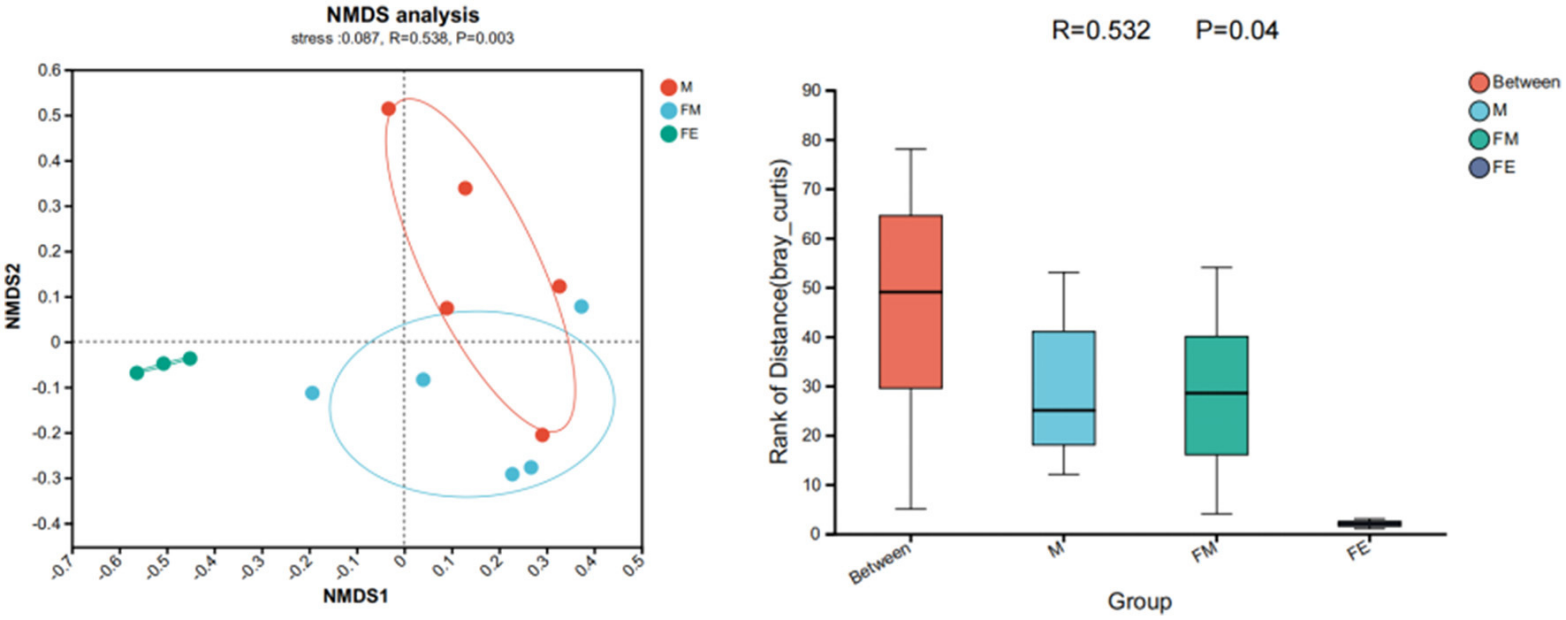
Differences in gut microbiota structure of *Erinaceus amurensis* based on NMDS (Non-metric multidimensional scaling) analysis and ANOSIM (Analysis of similarities) analysis. FM, female; M, male; FE, fetus.

The NMDS results reveal a stress value of 0.087, which is < 0.2, suggesting that the experimental findings possess explanatory significance. With the exception of the FE group, both other sample groups exhibit a relatively discrete mixed state. This implies that the gut microbiota structure between the male and female groups in the Wandashan Mountain range area are analogous, albeit with some variation from the fetal *Erinaceus amurensis*. This finding aligns with the ANOSIM analysis results, *R* = 0.532 > 0 ≈ 1, *P* = 0.004 < 0.05, indicating that there are disparities in the gut microbiota structure among the three groups. Furthermore, the intergroup differences surpass the intragroup differences (Figure 6).

### 3.3 Differences in dominant gut microbiota groups between different gender and adult-fetus pairs of the *Erinaceus amurensis* in the Wandashan Mountain range area

The phylogenetic analysis reveals that the gut microbiota samples from the three groups of *Erinaceus amurensis* exhibit distinct dominant bacterial community structures. Specifically, Group M demonstrates a more abundant dominant phylum, followed by Group FM, while Group FE presents the most sole dominant phylum. At the phylum level, *Pseudomonadota* dominates in Group FE, followed by both *Pseudomonadota* and *Bacteroidota* in Group FM, and *Pseudomonadota, Bacillota*, and *Bacteroidota* in Group M. At the class level, *Gammaproteobacteria* is the dominant class in Group FE.

**Figure 7:**
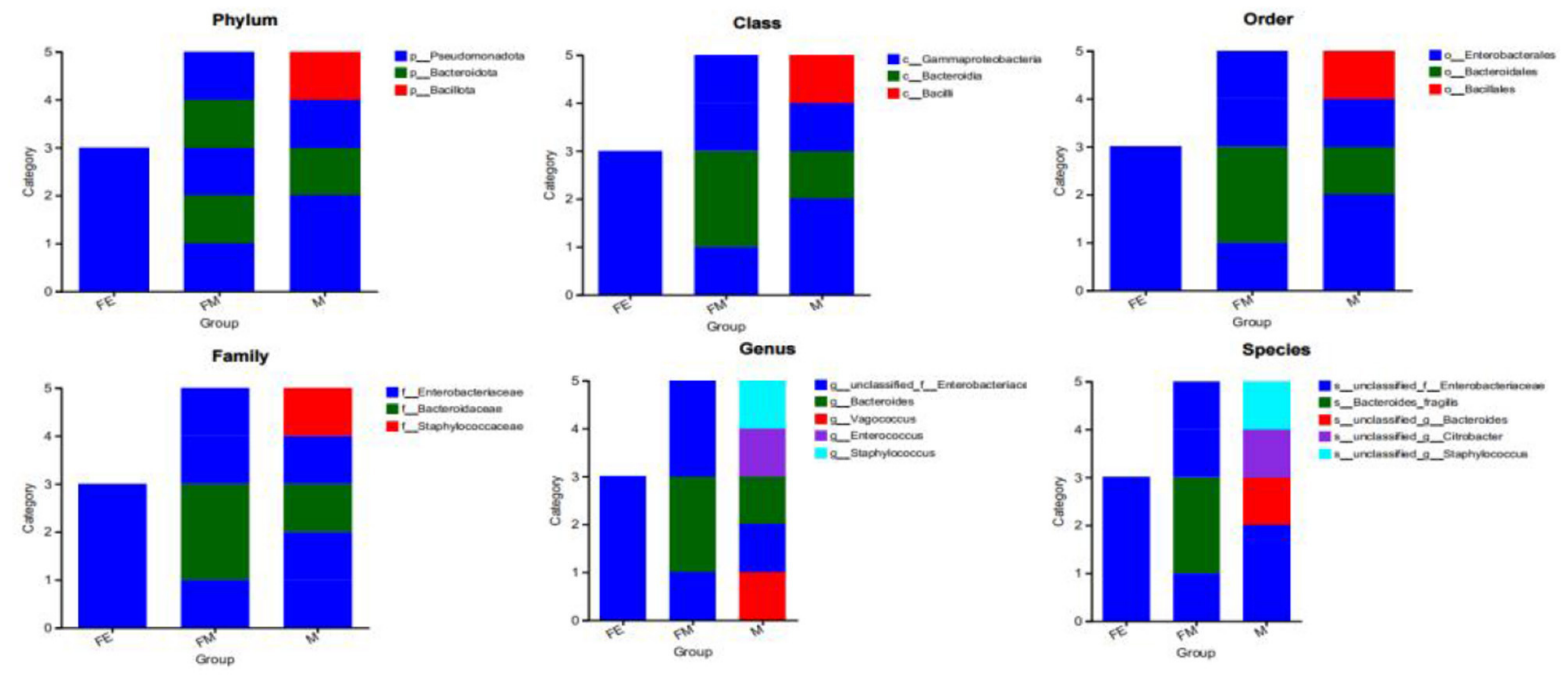
Distribution of three dominant bacterial groups at different classification levels. FM, female; M, male; FE, fetus.

In the FM group, the predominant bacterial classes are *Gammaproteobacteria* and *Bacteroidia*. Conversely, in the M group, the dominant bacterial phyla include *Gammaproteobacteria, Bacteroidia*, and *Bacilli*. At the order level, *Enterobacterales* are the most prevalent in the FE group, followed by *Enterobacterales* and *Bacteroidales* in the FM group, and *Enterobacterales, Bacteroidales*, and *Bacillales* in the M group. The dominant bacterial families at this level are *Enterobacteriaceae* in the FE group, followed by *Enterobacteriaceae* and *Bacteroidaceae* in the FM group, and *Enterobacteriaceae, Bacteroidaceae*, and *Staphylococcaceae* in the M group. At the genus level, unclassified_f__*Enterobacteriaceae* is the most prevalent in the FE group, followed by unclassified_f_*_Enterobacteriaceae* and *Bacteroidaceae* in the FM group, and unclassified_f__*Enterobacteriaceae, Bacteroidaceae, Vagococcus, Enterococcus*, and *Staphylococcus* in the M group. Finally, at the species level, *Enterobacteriaceae* is the most prevalent in the FE group, followed by *Enterobacteriaceae* and *Bacteroides*_fragilis in the FM group, and *Enterobacteriaceae, Bacteroides, Citrobacter*, and *Staphylococcus* in the M group (Figure 7).

## 4 Discussion

### 4.1 Composition of the gut microbiota in *Erinaceus amurensis*

The *Erinaceus amurensis* is the most widely distributed subspecies of the *Erinaceus* genus in the wild habitats within China, has garnered scientific interest due to its potential as an experimental animal and the unique value of its fur for traditional Chinese medicine research. The gut microbiota, a complex ecological community that establishes a dynamic balance within the animal's gut, plays a crucial role in maintaining the homeostasis of the gut microbiota and the health of the host animal. An imbalance in the gut microbiota can directly or indirectly impact the health level of the host animal, potentially posing a threat to its survival (Glenny et al., 2017). In this study, we conducted metagenomic sequencing on fecal samples from 13 *Erinaceus amurensis* in the Wandashan Foothills area. Our aim was to understand the composition, abundance, and structure of the gut microbiota among different genders, adults, and fetuses. This data will provide foundational information for further research on the protection of wild *Erinaceus amurensis* populations and their use as experimental animals. This, in turn, will promote advancements in comparative medicine and experimental animal studies.

In phylum-level classification, the *Pseudomonadota* phylum is the most prevalent in three distinct sample groups. *Pseudomonas*, a type of Gram-negative rod-shaped bacteria, is ubiquitously found in various environments including soil, plant surfaces, and within organisms. This bacterium exhibits potent antibacterial activity on plant surfaces and is frequently employed to enhance crop growth. Furthermore, it demonstrates significant antibiotic resistance within organisms. In a 2019 report on the dangers of antibiotic resistance, the U.S. CDC highlighted that *Pseudomonas* exhibits near-complete resistance to all antibiotics, inclusive of carbapenems (CDC, 2019). This aligns with the geographical environment of the experimental animal's acquisition site, which is situated at the intersection of forest and farmland in a major agricultural county. In recent years, the frequent use of antibiotics at concentrations significantly exceeding therapeutic doses in agricultural production to enhance crop growth has resulted in widespread antibiotic residues within this region's food chain. Consequently, there has been a significant increase in the number of antibiotic-resistant bacteria within the gut microbiota of wild animals.

The content of *Pseudomonas* in the three groups of *Erinaceus amurensis* samples is roughly the same, with the FE group being newborns that have not yet been breastfed and have not engaged in other predatory activities. Studies have found that fetuses begin to come into contact with and acquire microbes in the mother's womb, and factors affecting the establishment and dynamic changes of gut flora in juvenile individuals from birth to weaning include modes of delivery, Maternal and paternal transmission, food composition, and use of antibiotics (Dubois et al., 2024; Wu et al., 2024; Laborda et al., 2022; Aagaard et al., 2014). Based on these research findings, there are several possibilities: Horizontal transmission: The fetus may acquire resistance genes by coming into contact with bacteria on the mother's skin or in the surrounding environment, or it may be infected during the birth process through the mother's birth canal; Vertical inheritance/mother-infant transmission: Resistance genes may be passed from the mother to the fetus. During pregnancy, the mother's resistance genes can be transferred to the fetus through the uterus, and bacteria can be transmitted between the mother and fetus through blood circulation; Transmission of commensal bacteria: the mother's commensal bacteria may rapidly colonize after the fetus is born through methods such as licking the fetus's mouth and nose. These commensal bacteria may carry resistance genes and spread between mother and infant through contact. Which specific mode of transmission and mechanism of transmission needs further study.

The *Bacillota* and *Bacteroidota* are the predominant bacterial groups in the *Erinaceus amurensis*, mirroring the dominant bacterial groups in the gut microbiota of typical ungulates, rodents, and primates (Scott et al., 2013; Delgado et al., 2017; Costa et al., 2012; Menke et al., 2014; Haigh et al., 2014). Despite being a small mammal from the Insectivora order, research has shown that the *Erinaceus amurensis* also exhibits an omnivorous diet (Waite and Taylor, 2014). The *Bacillota* possesses robust degradation capabilities, aiding the host in breaking down food fibers to facilitate polysaccharide degradation, thereby enhancing energy acquisition from food (Gharechahi et al., 2023). The *Bacteroidota* plays a role in degrading carbohydrates and proteins, thus aiding the host in improving nutritional efficiency and promoting digestion and absorption of food (Shin et al., 2024). The comparable abundance of *Bacillota* and *Bacteroidota* between group M and group FM suggests that the gut microbiota of male and female individuals share similar functions in fiber, carbohydrate, and protein degradation. However, the lower abundance of *Bacteroidota* in group FE compared to adult groups M and FM indicates that the fetus's capacity for carbohydrate and protein degradation is less than that of adults.

At the genus level, *Escherichia*, a typical mammalian microbiota, can colonize the intestine and inhibit the colonization of potential pathogenic microorganisms. However, certain strains may acquire pathogenic genes during evolution, resulting in diseases such as urethritis, cystitis, diarrhea, and urinary tract infections in both humans and animals (Nasrollahian et al., 2024; Abad-Fau et al., 2024). The gut enrichment level of *Escherichia* coli is notably higher in the FM group compared to the M group, whereas the FE group exhibits the lowest level of enrichment. The genus *Salmonella* is responsible for causing salmonellosis, a prevalent foodborne zoonotic disease that presents a significant global public health challenge. Infection symptoms encompass nausea, vomiting, acute enterocolitis with accompanying abdominal pain, and diarrhea, which may or may not be bloody (Trees et al., 2024; Anderson et al., 2017). This disease can proliferate in the intestinal tracts of animals through various vectors, including the consumption of contaminated food, infection due to compromised immunity in animals, or transmission via environmental mediums such as soil or water sources. The intestinal enrichment levels of Salmonella in the FE group exceed those in the M group, while the FM group exhibits the least enrichment. The *Staphlococcus* is widely recognized as an animal pathogen, having been isolated from pets, livestock animals, and wildlife. It frequently acts as an opportunistic pathogen in several serious infectious diseases such as sinusitis, endocarditis, peritonitis, septic shock, urinary tract infections, and wound infections. Typically, it is more susceptible to newborns or populations with relatively low immunity due to severe underlying diseases (Hay and Sherris, 2020; Ortega-Peña et al., 2019). The results of this experiment confirmed that the abundance of *Staphylococcus* in the fetal FE group was higher than that in the adult M and FM groups.

Furthermore, *Klebsiella, Morganella*, and *Psychrobacter* exhibit specific pathogenicity that can induce symptoms such as wound infection in the host, Pneumonia, diarrhea, and urinary tract infections. The bacterial communities identified in these samples were enriched across all three experimental groups, indicating a potential correlation with intestinal diseases and urinary tract infections among the *Erinaceus amurensis* of the Wandashan Mountain range area. Notably, the FM group exhibited the least degree of pathogen enrichment compared to the other two groups. This could be attributed to the timing of sampling, which coincided with the gestation or parturition period for female *Erinaceus amurensi*s. The primary methods of *Morganella* enrichment in the animal intestine are fecal-oral transmission, sexual contact, and vertical transmission from mother to offspring. Research indicates that mammals establish their intestinal flora by ingesting the mother's feces immediately after birth. If the mother is infected with *Morganella*, it can result in an infection within the uterine cavity, thereby transmitting the bacteria to the fetus. Furthermore, during mating, if one partner is infected with this bacterium, it can be transmitted through the mucous membranes of the genitals. *Klebsiella*, on the other hand, spreads in the animal intestine through airborne contact or aerosols, consistent with the development of non-ferrous metals minerals in the sample collection area. These bacteria are enriched in all three experimental groups. These findings suggest that hedgehogs in the northeastern region of the Wanda Mountains may be susceptible to related intestinal diseases, pneumonia, and urinary tract infections. The bacterial communities were found to be enriched in all three experimental groups. This finding further implies a potential correlation between intestinal diseases, pneumonia, and urinary tract infections in the Northeast hedgehogs from the Wandashan area. Notably, the pathogenic bacteria in female FM exhibited the lowest enrichment levels among the three groups. This could potentially be attributed to the sampling time coinciding with the pregnancy or birthing period of the female *Erinaceus amurensis*. Previous research has demonstrated that antibacterial and anti-inflammatory substances are produced in the intestines during pregnancy, serving to enhance the survival capabilities of female animals in their natural habitat (Santos et al., 2020).

The *Caudoviricetes* plays a crucial role in maintaining the homeostasis of the gut microbiota in mammals. Bacteriophages, which attack host bacteria, can limit bacterial behavior and pathogenicity, thereby contributing to the construction of a healthy microbial structure for the host (Norman et al., 2015; Manrique et al., 2016). In the three groups studied, the abundance of Caudoviricetes was higher in the adult M group and FM group than in the fetal FE group. This suggests that the gut microbiota repair and homeostasis maintenance capabilities are stronger in adult *Erinaceus amurensi*s than in fetal ones.

### 4.2 Diversity differences in the gut microbiota of *Erinaceus amurensi*s

Through Alpha diversity analysis of the gut microbiota in three groups of samples, and based on NMDS and ANOSIM analyses, it was found that there are certain differences in the diversity of gut microbiota among the three groups: Among them, the microbial diversity in group M is higher than that in group FM, and the lowest is in group FE; Differences in gut microbiota structure among three groups: significant differences between adults and fetuses. Jiang et al. (2022) research suggests that the diversity and richness of the microbiota in male *Moschus berezovskii* exceed those in females. This contradicts the previously held belief that “the richness and diversity of gut microbiota are typically greater in females than in males,” Naturally, variations in background factors also play a role, including age, dietary habits, seasonal factors, geographical location, and health status (Fang et al., 2024; Jiang et al., 2020). Hong Yang's study on the gender-based diversity of gut microbiota in *Ailuropoda melanoleuca* revealed a correlation between age and the observed differences. Specifically, the gut microbial community diversity in subadult and adult female *Ailuropoda melanoleuca* exceeds that of their male counterparts. Conversely, elderly female *Ailuropoda melanoleuca* exhibit lower gut microbial community diversity compared to males (Benler et al., 2021).

From April to July, during the transition from spring to summer, the dietary options for the *Erinaceus amurensis* diversify due to seasonal variations. This observed phenomenon in our study may be attributed to the fact that the sampling period aligns with the female breeding and lactation season. During this time, female animals tend to limit their hunting range to care for and safeguard their offspring. This behavior results in temporary alterations in their prey selection and feeding habits, which in turn could influence the diversity of their gut microbiota. Conversely, male *Erinaceus amurensis* strive to maximize their territory during this season, engaging in frequent hunts to accumulate energy and ensure species survival. Consequently, their activity radius is broader than that of the females, leading to a more varied diet and, subsequently, a greater diversity in their gut microbiota compared to their female counterparts.

The FE group exhibited the least diversity in gut microbiota among the three groups. This could potentially be attributed to mammals briefly existing in a sterile environment immediately post-birth, or due to a lower bacterial count. However, as bacteria begin to colonize and establish themselves within the gastrointestinal tract, a gut microbiota can develop over ~12 months. Consequently, under favorable conditions, various types of exogenous microorganisms penetrate the gut and colonize, thereby enhancing the diversity of the gut microbiota. Over time, dominant bacterial populations become more pronounced, eventually stabilizing within the gut, Studies have demonstrated that a decrease in both systemic diversity and abundance of animal gut microbiota correlates with diminished resistance and structural stability within the organism (Yan et al., 2021; Schnupf et al., 2018). This aligns with the findings of previous research, which identified a diverse array of pathogenic bacteria within the gut microbiota of the FE group. Furthermore, these bacteria were found in greater abundance than those in both the M and FM groups, as the fetus develops, beneficial bacteria progressively colonize the intestinal tract, possessing the capacity to eradicate or suppress pathogenic bacteria.

### 4.3 Differences in dominant gut bacterial communities of *Erinaceus amurensi*s

The microbial community typing analysis was conducted to ascertain the dominant bacterial community structure in three distinct sample groups. *Gammaproteobacteria* can assist the host in utilizing carbon sources from food and store energy from food for the host. *Enterobacterales*, as a genus under the class *Proteobacteria*, also has functions such as synthesizing vitamins, promoting gastrointestinal motility, accelerating digestion and absorption in the intestine, and controlling metabolism (Lu et al., 2012), both and were widely present in the three groups of samples, the *Erinaceus amurensi*s exhibits a hibernating behavior, with the hibernation period spanning from October to March of the subsequent year, totaling 6 months. The gut of this species harbors two distinct bacterial communities that play crucial roles in energy storage and metabolic regulation, thereby facilitating its successful survival during hibernation. The *Bacillota* can provide energy supply for the host (Furet et al., 2009). *Enterococcus*, which serve as intestinal symbiotic bacteria, are capable of producing organic acids and bacteriocins, among other antimicrobial substances. These substances inhibit the reproduction of pathogenic bacteria and contribute to the host animal's immune regulation, thereby enhancing the body's immunity (Krawczyk et al., 2021). Within Group M, *Bacillota* and *Enterococcus* are the predominant bacterial groups. This dominance could be attributed to the timing of the sampling, which coincided with the mating and breeding season for the *Erinaceus amurensi*s. Male *Erinaceus amurensi*s exhibit territorial behavior and must accumulate more energy and maintain superior physical fitness to compete with other males within their domain. They strive to maximize their territory size to secure additional mating opportunities or to mate more frequently for reproductive purposes. These physiological activities necessitate a substantial energy reserve and robust physical fitness. Bacteria within the *Bacteroidota* have the capacity to produce an increased amount of propionate, which in turn stimulates gluconeogenesis. This biochemical process in the liver enables the host animal to experience a sense of satiety (Morrison and Preston, 2016). In the three sample groups, *Bacteroidota* was the dominant bacterial group in both Group M and Group FM, while Group FE showed no distribution. This may be attributed to the fact that juvenile animals are breastfeeding by their mothers for a period post-birth, thereby providing a consistent food source and essentially maintaining a “full” state. Conversely, adult individuals encounter various survival pressures in nature, and instability in food sources can sometimes result in feelings of hunger. The *Bacteroidota* aids adult *Erinaceus amurensi*s in resisting hunger in the short term prior to obtaining food, thereby enhancing their survival capabilities in nature. *Citrobacter*, a common inhabitant of the animal gut (Brenner et al., 1999), is ubiquitously distributed in soil, water, air, and food. It also serves as a prevalent pathogenic bacterium exhibiting robust drug resistance, capable of impacting both human and animal health. Infections induced by *Citrobacter* can result in a range of diseases, including skin and soft tissue infections, urinary tract infections, pneumonia, and sepsis in animals. Following infection, animals frequently suffer from respiratory distress or sepsis, which can be fatal in extreme cases (Xiao et al., 2021; Fonton et al., 2024; Gill and Schutze, 1999). *Citrobacter* was the predominant bacterial genus in group M, but not in the other two groups. This suggests that male *Erinaceus amurensi*s in this region may be afflicted with associated diseases, necessitating measures for their wild rescue and protection.

## 5 Conclusion

This study undertook an initial examination and investigation of the composition and diversity of the intestinal microbiota of the *Erinaceus amurensi*s in the Wandashan area from April to July, utilizing metagenomic sequencing technology. The findings indicate distinct differences in the intestinal microbiota of the *Erinaceus amurensi*s across genders, adults, and fetuses. Specifically, the predominant bacterial communities within the intestines of the *Erinaceus amurensi*s primarily consist of *Pseudomonas, Proteobacteria*, and *Enterobacteriaceae*. At the phylum level, male *Erinaceus amurensi*s exhibit one additional dominant intestinal bacterial group compared to their female counterparts, and two more than those found in fetal dominant bacterial groups. The predominant bacterial groups identified in both male and female *Erinaceus amurensi*s are *Pseudomonas, Bacteroides*, and *Firmicutes*. Notably, there are significant differences in microbial abundance and diversity, with males demonstrating greater quantities than females. While the overall composition of microbes remains similar, distinct variations are observed within different bacterial classifications. The dominant bacterial community in fetal *Erinaceus amurensi*s is *Gammaproteobacteria*, which displays reduced diversity and abundance compared to the adult cohort. Furthermore, the types and prevalence of pathogenic or opportunistic pathogens in the gut of fetal *Erinaceus amurensi*s and males surpass those found in females. Consequently, it is advised that forestry and wildlife conservation professionals in this region prioritize the prevention and treatment of inflammation associated with the gut, urethra, and skin during the rescue of wild *Erinaceus amurensi*s. This research serves as a foundation for future studies on the determinants influencing the gut microbiota of the *Erinaceus amurensi*s, including the diversity and classification of microbiota across various intestinal segments. Furthermore, it delves into the functionalities of the gut microbiota, antibiotic resistance genes, and the presence of intestinal microplastics. Such insights are pivotal for the conservation and rehabilitation of the wild *Erinaceus amurensi*s population and offer valuable contributions to experimental animal physicochemical investigations.

## Data Availability

The data presented in the study are deposited in the NCBI repository, accession number PRJNA1146738.
